# Gluconolactone Alleviates Myocardial Ischemia/Reperfusion Injury and Arrhythmias *via* Activating PKCε/Extracellular Signal-Regulated Kinase Signaling

**DOI:** 10.3389/fphys.2022.856699

**Published:** 2022-03-14

**Authors:** Xinghua Qin, Binghua Liu, Feng Gao, Yuanyuan Hu, Ziwei Chen, Jie Xu, Xing Zhang

**Affiliations:** ^1^School of Life Sciences, Northwestern Polytechnical University, Xi’an, China; ^2^Shaanxi Engineering Laboratory for Food Green Processing and Safety Control, College of Food Engineering and Nutritional Science, Shaanxi Normal University, Xi’an, China; ^3^Research Center for Prevention and Treatment of Respiratory Disease, School of Clinical Medicine, Xi’an Medical University, Xi’an, China; ^4^Department of Cardiology, 986th Hospital, Fourth Military Medical University, Xi’an, China; ^5^School of Aerospace Medicine, Fourth Military Medical University, Xi’an, China

**Keywords:** gluconolactone, Ischemia/Reperfusion injury, PKC, ERK, ROS

## Abstract

Gluconolactone (D-glucono-1,5-lactone or GDL) is a food additive which presents in dietary products such as tofu, yogurt, cheese, bread, wine, etc. GDL has long been considered as a free radical scavenger; however, its role in cardioprotection remains elusive. In this study, using a mouse model of myocardial ischemia/reperfusion (I/R) injury and a model of hypoxia/reoxygenation (H/R) in neonatal rat cardiomyocytes (NRCM), we explored the role of GDL in I/R injury. We found that GDL (5 mg/kg, i.p.) attenuated myocardial I/R injury as evidenced by decreased infarct size, release of cardiac injury markers and apoptosis. Additionally, GDL decreased reperfusion-induced arrhythmias and oxidative stress. These effects were also observed in parallel *in vitro* studies. Mechanistically, we found that GDL treatment was strongly associated with activation of pro-survival extracellular signal-regulated kinase (ERK) signaling both *in vivo* and *in vitro*, and pharmacological inhibition of ERK signaling *via* U0126 attenuated GDL-induced cardioprotection against H/R injury in NRCM cells. To reveal how GDL regulates ERK signaling, we predicted the putative targets of GDL by Swiss Target Prediction, and protein kinase C (PKC) emerged as the most promising target for GDL. By pharmacological intervention and immunofluorescence, we found that PKCε, an important member of the PKC family, was activated after GDL treatment in heart, thereby leading to ERK activation and cardioprotection against I/R injury. Taken together, our results demonstrated that GDL acts as a potent activator of PKCε and, thus, provides cardioprotection against I/R injury *via* activation of ERK signaling.

## Introduction

Gluconolactone (D-glucono-1,5-lactone or GDL) is a polyhydroxy acid (PHA), a lactone or oxidized derivative of glucose, and is widely distributed in nature ranging from bacteria to humans. GDL can be naturally found in honey, grapes and fruit juices, and widely used in dietary products such as yogurt, cheese, bread, wine and tofu, etc., as a food additive (European food additive number E575). Recently, GDL as a human natural metabolite has attracted more and more attention, and its levels were shown to change significantly under different pathophysiological conditions. Zhao et al. (2018) demonstrated that fecal GDL increased about twofold in normal human subjects after half-marathon race compared with those before race. Makowski et al. (2014) showed that threefold decrease of GDL were observed in tumors from obese mice compared with lean counterparts. However, the role of GDL in the development of cardiovascular diseases remains elusive.

Ischemia/reperfusion (I/R) injury is a common pathological process in the clinic, and is associated with adverse postoperative outcomes. Reactive oxygen species (ROS) burst (superoxide radicals or hydrogen peroxide) at the onset of reperfusion is considered as one of the key factors contributing to reperfusion injury. In the presence of metals such as iron and copper, superoxide radicals or hydrogen peroxide could produce the most reactive ROS, the hydroxyl radical (•OH), thus aggravating the oxidative stress (Wu and Cederbaum, 2003). GDL, a natural PHA, is capable of chelating metals as an antioxidant chelating agent, and provides protection up to 50% against UV radiation (Bernstein et al., 2004; Bowes, 2013; Audina, 2021). Moreover, GDL could maintain the redox balance *via* increasing NADPH generation through entering the gluconate pathway and subsequent pentose-phosphate pathway (PPP; Wushensky et al., 2018), and was thus used as a probe to detect PPP flux (Moreno et al., 2017). These results indicated that GDL might exert cardioprotection against I/R injury *via* decreasing oxidative stress.

Evidence has shown that lactone exerts protective effects against various pathological conditions, including myocardial infarction and I/R injury. Wang et al. (2018) have shown that lactone component from *Ligusticum chuanxiong* significantly reduced infarct size in myocardial ischemia injured rats and H9c2 cardiomyocytes, probably due to the restoration of autophagic flux through activation of PI3K/Akt/mTOR signaling pathway. Moreover, 10 kinds of diterpene lactone compounds including Ginkgolide B (GB), Ginkgolide A (GA), and Ginkgolide K (GK) have been isolated from *Ginkgo biloba leaves*, and some of them are reported to provide the cardiocerebral protection (reviewed by Geng et al., 2018; Liu et al., 2019 in rat brain). Regarding I/R injury, lactones, such as ginkgo diterpene lactones, parthenolide, etc., have been reported to attenuate reperfusion injury in brain (Li et al., 2020), heart (Zingarelli et al., 2002; Liu et al., 2011) and PC12 cells (Zhang et al., 2017). Thus, it is easily to postulate that GDL, a δ-lactone formed by an inner ester of gluconic acid, might have the same effect like other lactones. GDL is a special lactone, as it is a human natural metabolite showing anti-oxidant properties, and revealing of its role in cardioprotection will help to discover new disease pathogenesis and find possible therapeutic targets.

In this study, using a mouse model of I/R injury and a cellular model of hypoxia/reoxygenation (H/R) mimicking I/R injury, we try to explore the role of GDL in I/R injury and the underlying mechanism. Our results demonstrated that GDL exerted cardioprotection against I/R injury, partly due to the activation of its potential target PKC and downstream pro-survival extracellular signal-regulated kinase (ERK) signaling.

## Materials and Methods

### Materials and Chemicals

D-glucono-1,5-lactone was obtained from MedChem Express (Princeton, NJ, United States). Triphenyltetrazolium chloride (TTC), PI3K inhibitor Wortmannin (Wm; inhibition of PI3K/Akt signaling) and mitogen-activated protein kinase kinase (MEK) inhibitor U0126 (inhibition of MEK/ERK signaling) were purchased from Sigma (St. Louis, MO, United States). PKC inhibitors Chelerythrine and Rottlerin were purchased from Topscience (Shanghai, China). PKC inhibitor Gö6976 was purchased from Beyotime (Haimen, China). PKCεV1-2 was obtained from Medbio (Shanghai, China). Dihydroethidium (DHE) was purchased from Beyotime.

Cell Counting Kit-8 (CCK-8) was obtained from Topscience. Lipid Peroxidation Malondialdehyde (MDA) Kit and Total Superoxide Dismutase (SOD) Assay Kit were purchased from Beyotime. *In Situ* Cell Death Detection Kit was purchased from Roche Co. (Mannheim, Germany). Lactate Dehydrogenase (LDH) Assay Kit, Creatine Kinase MB (CK-MB) isoenzyme Assay Kit and Coenzyme II (NADPH/NADP^+^) Content Test Kit were purchased from Nanjing Jiancheng Bioengineering Institute (Nanjing, China).

### Animals

Age-matched male C57BL/6J mice (8- to 10-week-old) were provided by the Experimental Animal Center of the Fourth Military Medical University (Xi’an, China). All animals were kept at controlled environmental conditions (22 ± 2°C with a 12 h light/dark cycle), and allowed free access to food and water. All experiments were performed in strict accordance with the recommendations in the Guide for the National Institutes of Health Guidelines for the Use of Laboratory Animals (Publication No. 85–23, revised 1996), and approved by the Animal Care Committee of Northwestern Polytechnical University (Xi’an, China). The mice were allowed to adapt to the new conditions for 1 week before being used in this experiment.

### Myocardial Ischemia/Reperfusion and Electrophysiological Examinations

The mouse model of myocardial I/R injury was established by occluding the left anterior descending coronary artery (LAD) for 30 min followed by reperfusion for 120 min. Briefly, mice were anesthetized by pentobarbital sodium (60 mg/kg) after fasted overnight. After endotracheal intubation, mouse heart was exposed between the third and fourth ribs, and LAD was ligated with a 7-0 silk suture. After 30 min of ischemia, the slipknot was released, and myocardium was reperfused for 120 min. Mice in the sham group underwent the same surgical procedures, but without ligation. GDL (5 mg/kg) or its control vehicle (saline) was intraperitoneally administrated at 10 min pre-reperfusion. Surface lead II electrocardiogram (ECG) was continuously recorded during I/R by a physiologic signal-acquisition system (RM6240; Chengdu instrument factory, Chengdu, China), and the arrhythmia incidence and score were assessed according to the Lambeth Conventions (Curtis et al., 2013).

### Measurement of Infarct Size

The myocardial infarct size was evaluated by TTC-Evans blue double staining. After 2 h of reperfusion, the LAD was re-tied to stop the blood flow, and 1 mL of 2% Evans blue dye (Beijing Solarbio Science & Technology Co., Ltd., Beijing, China) was infused into the hearts *via* the ascending aorta. After that, the heart was sliced into 1 mm sections, and incubated with 1% TTC at 37°C for 20 min. Finally, the slices were store in 4% paraformaldehyde solution before digitally photographed. The area of left ventricle (LV), the area at risk (AAR), and infarct size (INF) were assessed with FIJI (ImageJ) software (National Institutes of Health, United States).

### Evaluation of Apoptosis

At the end of reperfusion, cardiac apoptosis was evaluated by the Terminal deoxynucleotidyl transferase dUTP nick end labeling (TUNEL) assay using the *In Situ* Cell Death Detection Kit (Roche) according to the manufacturer’s instructions.

### Isolation of Neonatal Rat Cardiomyocytes

Neonatal rat cardiomyocytes (NRCM) were isolated from the hearts of 1- to 3-day-old Sprague–Dawley rats. Briefly, rat hearts were removed, minced and digested with 0.1% Type II collagenase and 0.25% Trypsin. Cardiomyocytes were isolated by differential detachment and verified under the microscope. Isolated NRCM were cultured in high glucose Dulbecco’s modified Eagle’s medium (DMEM) supplemented with 10% fetal bovine serum (FBS) and 1% penicillin/streptomycin solution in a humidified 5% CO_2_ incubator (Boxun, Shanghai, China) at 37°C.

### Hypoxia/Reoxygenation

A cellular model of H/R was established to mimic myocardial I/R injury *in vitro*. Briefly, hypoxia was induced by incubation of NRCM cells with glucose-free and serum-free DMEM medium in an anaerobic chamber (95% N_2_, 5% CO_2_) at 37°C for 12 h. To mimic reperfusion, the hypoxic NRCM cells were cultured in serum-free DMEM medium (with 5.5 mM glucose) under normoxic conditions for 2 h at 37°C.

D-glucono-1,5-lactone (1 μM) was administrated before the onset of reoxygenation. To investigate whether blocking PI3K/Akt signaling or MEK/ERK signaling could attenuate GDL-conferred cardioprotection against H/R, ERK inhibitor U0126 (10 μM) or PI3K inhibitor Wm (100 nM) administrated concurrently with GDL before the onset of reoxygenation.

### Immunoblotting

At the end of reperfusion or reoxygenation, heart tissue or cell culture was collected, and proteins were isolated using radio-immunoprecipitation assay (RIPA) lysis buffer containing protease inhibitor cocktail (Roche), and subsequently quantitated by BCA kit (Pierce Chemical Company, Rockford, IL, United States). Equal amount of proteins were loaded and separated by 10 or 12% polyacrylamide gel electrophoresis (SDS-PAGE), and transferred to polyvinylidene difluoride (PVDF) membranes. The membranes were blocked with 5% skim milk for 1 h, and subsequently incubated with primary antibodies against p-ERK (1:1000; Cell Signaling Technology/CST, Danvers, MA, United States), ERK (1:1000; Proteintech, Wuhan, China), p-Akt (1:1000; CST), Akt (1:1000; CST), Bcl2 (1:1000; Proteintech), Bax (1:1000; Proteintech), Caspase3 (1:1000; Proteintech), The nuclear factor erythroid 2 related factor 2 (NRF2; 1:1000; Proteintech), SOD2 (1:1000; Proteintech) and PKCε (1:1000; Proteintech) overnight at 4°C. Subsequently, the membranes were incubated with appropriate secondary antibodies for 1–2 h. Finally, the blots were visualized by Tanon imaging system (Tanon Science & Technology Co., Ltd., China) and quantified by densitometry using Quantity One software (Bio-Rad, Hercules, CA, United States).

### Immunofluorescence

Cardiac tissues were fixed overnight with 4% paraformaldehyde, embedded in paraffin, and sectioned with a microtome in 5 μm slices. Myocardial slices were stained with PKCε antibodies (Proteintech), and photographed by an optical microscope (Nikon, Melville, NY, United States). For the colocalization analysis, Manders’ coefficient was calculated using Coloc2 plugin in FIJI (ImageJ) image processing software.^1^

### Statistical Analysis

All data were presented as means ± SEM and performed using Graph Pad Prism 8.0 software. Student’s *t*-test or Chi-square test and Fisher’s exact test (for arrhythmia incidence) was used to determine the significant difference among two groups, while one-way ANOVA with Bonferroni’s *post hoc* test was used among more than two groups. Statistical significance was accepted at *P* < 0.05.

## Results

### D-Glucono-1,5-Lactone Exerts Cardioprotection Against Myocardial Ischemia/Reperfusion Injury

**FIGURE 1:**
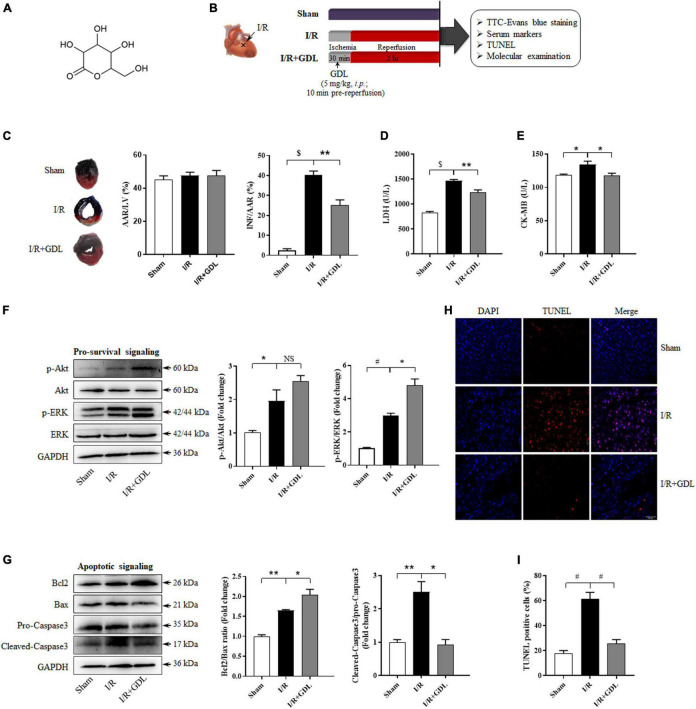
D-glucono-1,5-lactone protected against myocardial ischemia/reperfusion injury in mice. **(A)** The chemical structure of GDL. Mice were randomly divided into three groups: Sham, I/R group, and I/R + GDL group. **(B)** The schematic of the experimental design *in vivo*. **(C)** Myocardial infarction was assessed by TTC-Evans blue double staining (*n* = 5). **(D)** Serum LDH and **(E)** CK-MB activities were evaluated as an index for cardiac injury *via* commercial kits (*n* = 8–12). **(F)** Representative images and Western blot analysis of pro-survival kinases (ERK and Akt) in cardiac tissues of each group (*n* = 5). **(G)** Representative images and Western blot analysis of apoptosis-related proteins (Bcl2, Bax, and Caspase3) in cardiac tissues of each group (*n* = 5). **(H–I)** TUNEL staining and quantification of apoptosis in heart tissue section among groups (*n* = 5; scale bar = 50 μm). *P < 0.05, **P < 0.01, ^#^*P* < 0.001, and ^$^*P* < 0.0001 between indicated groups.

D-glucono-1,5-lactone, a derivate of glucose, is a ring-shaped molecule characterized by a tetrahydropyran substituted by three hydroxyl groups, one ketone group, and one hydroxymethyl group (its chemical structure is displayed in Figure 1A). The schematic of the experimental design *in vivo* is shown in Figure 1B. GDL administration reduced myocardial infarction size in IR + GDL group (25 ± 3%) compared with I/R group (40 ± 2%) (Figure 1C). In line with this, serum levels of cardiac injury markers, LDH and CK-MB were increased following I/R injury, while were decreased with GDL pre-treatment (Figures 1D,E).

Imbalance between pro-survival and apoptotic signaling is believed to be central to myocyte dysfunction following I/R injury. Thus, we assessed the expression and (or) activity of key players in pro-survival (Akt and ERK) and apoptotic (Bcl2, Bax, and Caspase3) signaling pathways by Western blot analysis, demonstrating that GDL treatment stimulated ERK phosphorylation, especially p42 ERK, whereas no significant difference in Akt expression and phosphorylation was observed between I/R and I/R + GDL mice (Figure 1F). GDL administration following I/R decreased apoptotic signaling in cardiac tissues as evidenced by increased Bcl2/Bax ratio and decreased Caspase3 activation (Figure 1G). In agreement with this, the terminal deoxynucleotidyl transferase (TdT) dUTP nick-end labeling (TUNEL) staining showed that GDL decreased I/R-induced apoptosis in cardiac tissue (Figures 1H,I).

### D-Glucono-1,5-Lactone Protects Against Reperfusion-Induced Arrhythmias

**FIGURE 2:**
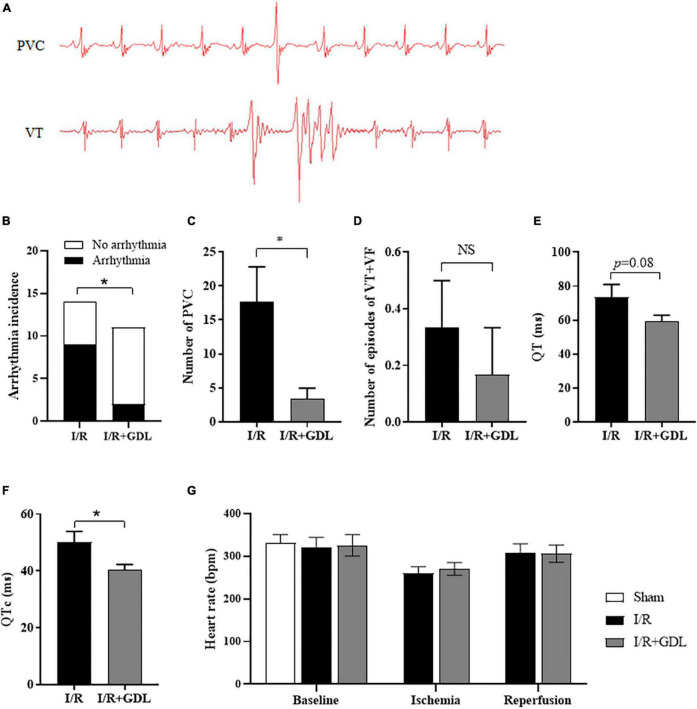
D-glucono-1,5-lactone reduced reperfusion-induced arrhythmias. **(A)** Representative examples of different ventricular arrhythmias (PVC and VT) during 30 min post-reperfusion. Arrhythmia incidence **(B)**, number of PVC **(C)**, number of VT + VF **(D)**, QT duration **(E)** and corrected QT **(F)** in I/R-injured mice in the presence or absence of GDL treatment (*n* = 6). Heart rate **(G)** of all sections, including baseline, ischemia and reperfusion (*n* = 6). *P < 0.05 between indicated groups.

Reperfusion-induced arrhythmia is a common clinical manifestation of myocardial I/R injury. The surface ECG was recorded in each group, and arrhythmias were analyzed 30 min post-reperfusion according to the Lambeth conventions (Curtis et al., 2013). ECG showed that arrhythmias were commonly seen during the onset of reperfusion, of which, mostly were premature ventricular contraction (PVC), rarely were ventricular tachycardia (VT), and none were ventricular fibrillation (VF). Representative examples of different ventricular arrhythmias (PVC and VT) were illustrated in Figure 2A. GDL decreased reperfusion-induced arrhythmias (Figure 2B), mainly due to a decrease of PVC but not VT occurrence (Figures 2C,D). Of note, we found that QT interval and corrected QT (QTc) interval, a risk factor for the development of ventricular arrhythmia, were decreased in mice with GDL treatment (Figures 2E,F). Heart rate showed no apparent changes during baseline, ischemia or reperfusion in I/R-injured mice with or without GDL treatment (Figure 2G).

### D-Glucono-1,5-Lactone Decreases Oxidative Stress Induced by Ischemia/Reperfusion

**FIGURE 3:**
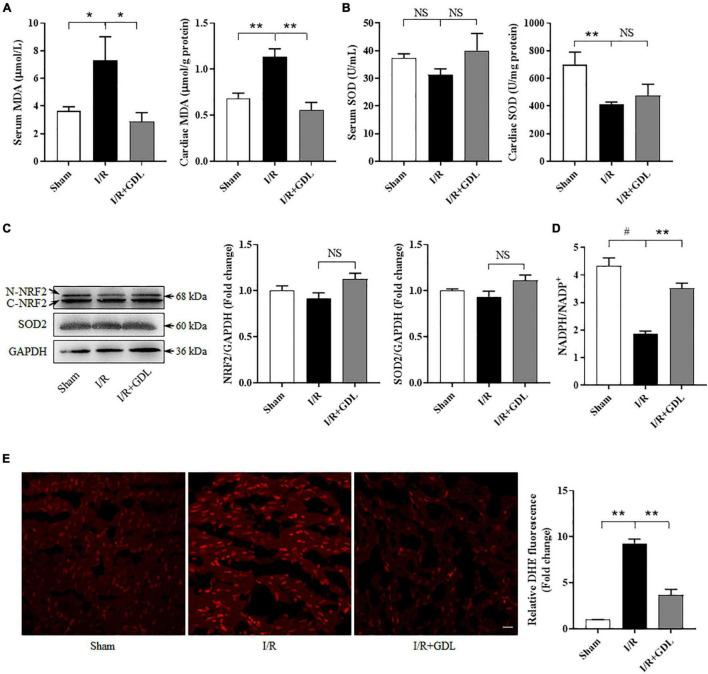
D-glucono-1,5-lactone attenuated reperfusion-induced oxidative stress. **(A)** MDA levels in serum and cardiac tissue in sham, I/R, and I/R + GDL groups (*n* = 4–6). **(B)** SOD activities in serum and cardiac tissue in sham, I/R, and I/R + GDL groups (*n* = 6–7). **(C)** Representative images and Western blot analysis of NRF2 and SOD2 expression in sham, I/R, and I/R + GDL groups (*n* = 5). **(D)** NADPH/NADP^+^ ratio among groups (*n* = 3–4). **(E)** DHE staining of heart tissue section among groups (*n* = 3; scale bar = 20 μm). *P < 0.05, **P < 0.01, and ^#^*P* < 0.001 between indicated groups. NS, not significant.

Although GDL is known as an antioxidant, its regulatory role in oxidative stress *in vivo*, especially in the context of I/R, was not investigated. As shown in Figures 3A,B, exposure to I/R displayed an increase of MDA and a decrease of SOD2 activity in either serum or heart tissue. GDL treatment decreased MDA levels both in serum and heart tissue of I/R-injured mice, yet no significant changes of SOD2 activity were observed after GDL treatment (Figures 3A,B).

Oxidative stress refers to the pathological process of tissue damage caused by excessive ROS production and/or reduced ROS scavenging capacity. We first evaluated the expression of antioxidant enzymes including NRF2 and SOD2. In line with the results of our studies regarding to SOD activities, no apparent changes of NRF2 and SOD2 expression were observed among groups (Figure 3C). NADPH, a key component in the cellular anti-oxidative defense system, was decreased in the heart of I/R mice, an effect which could be reversed by GDL treatment (Figure 3D). Next, we measured total ROS levels using DHE staining, showing that GDL treatment decreased I/R-induced ROS levels (Figure 3E).

### D-Glucono-1,5-Lactone Increases Cell Viability of Neonatal Rat Cardiomyocytes Cells Following Hypoxia/Reoxygenation Injury

**FIGURE 4:**
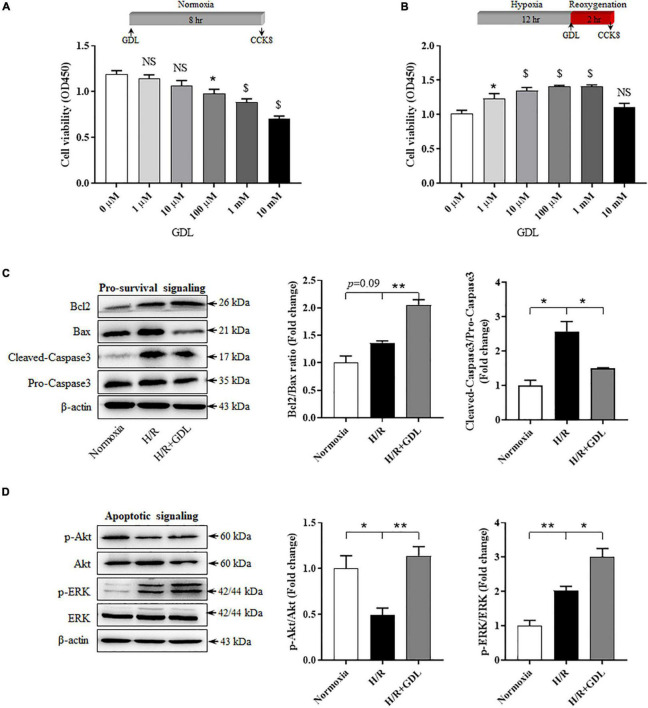
D-glucono-1,5-lactone enhanced cell viability of NRCM following H/R injury. **(A)** Dose-dependent effect of GDL (0 μM, 1 μM, 10 μM, 100 μM, 1 mM, and 10 mM) on cell viability of NRCM cells by CCK-8 test. **(B)** Dose-dependent effect of GDL (0 μM, 1 μM, 10 μM, 100 μM, 1 mM, and 10 mM) on cell viability of H/R-injured NRCM cells by CCK-8 test. **(C)** Representative images and Western blot analysis of apoptosis-related proteins (Bcl2, Bax, and Caspase3) in normoxia, H/R and H/R + GDL groups. **(D)** Representative images and Western blot analysis of pro-survival kinases (ERK and Akt) among groups. *P < 0.05, **P < 0.01, and ^$^*P* < 0.0001 between indicated groups. Data shown are representive of five independent experiments.

Next, we evaluated the protective effect of GDL and the underlying mechanism *in vitro*. Our results demonstrated that high concentration of GDL (>100 μM) had a cytotoxic effect on NRCM cells, whereas GDL concentrations at 1 μM and 10 μM did not show this adverse effect (Figure 4A). In the context of H/R injury, GDL concentrations at 1 μM, 10 μM, 100 μM and 1 mM showed beneficial effects against H/R injury, an effect that was diminished when GDL concentration was at 10 mM (Figure 4B). Therefore, GDL concentration in the following experiments was set to be 1 μM when GDL exerted cardioprotective effects against H/R injury and had less cytotoxic effect. As expected, GDL (1 μM) treatment decreased apoptotic signaling in H/R-injured NRCM cells, as evidenced by decreased Caspase3 activation and increased Bcl2/Bax ratio (Figure 4C). In agreement with this result, GDL treatment activated pro-survival signaling including ERK and Akt (Figure 4D).

### Inhibition of Extracellular Signal-Regulated Kinase Signaling *via* U0126 Attenuates D-Glucono-1,5-Lactone Protection Against Hypoxia/Reoxygenation Injury

**FIGURE 5:**
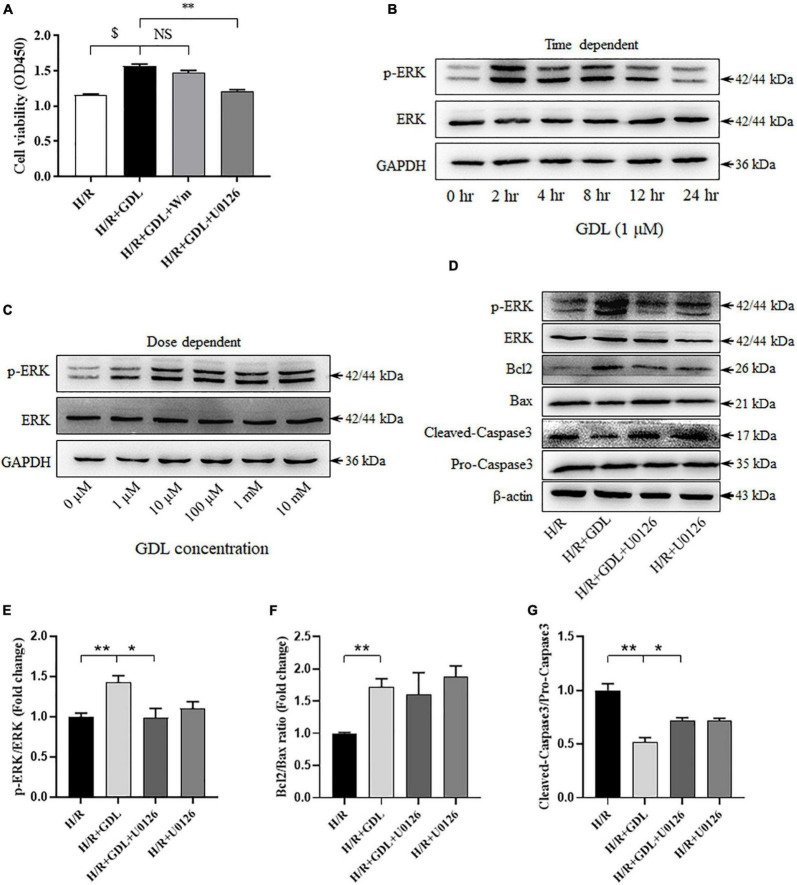
Extracellular signal-regulated kinase signaling contributed to GDL-conferred cardioprotection against H/R injury. **(A)** The effect of MEK/ERK inhibitor U0126 (10 μM) or PI3K/Akt inhibitor Wortmannin (Wm; 100 nM) on GDL-induced cardioprotection against H/R injury. **(B)** Time-dependent effect of GDL (0, 2, 4, 8, 12, and 24 h) on ERK expression and phosphorylation by Western blot analysis. **(C)** Dose-dependent effect of GDL (0 μM, 1 μM, 10 μM, 100 μM, 1 mM and 10 mM) on ERK expression and phosphorylation by Western blot analysis. **(D–G)** Representative images and Western blot analysis of apoptosis-related proteins (Bcl2, Bax, and Caspase3) and pro-survival ERK signaling in H/R, H/R + GDL, H/R + GDL + U0126, H/R + U0126 groups. *P < 0.05 and **P < 0.01 between indicated groups. NS, not significant. Data shown are representive of five independent experiments.

Both *in vivo* and *in vitro* studies have shown that GDL-conferred cardioprotection was associated with increased pro-survival signaling (ERK or Akt). CCK-8 assay showed that blocking ERK signaling by its inhibitor U0126 attenuated GDL-induced cell survival, whereas inhibiting PI3K/Akt signaling *via* Wortmannin did not show this effect (Figure 5A). Supportively, GDL treatment activated ERK signaling, reaching a peak at 2 h (Figure 5B). In addition, either low or high concentration of GDL strongly activated ERK signaling after treating cardiomyocytes for 2 h (Figure 5C). Western blot analysis showed that blocking ERK signaling attenuated GDL-induced inhibition of Caspase3, yet had no significant effect on Bcl2/Bax ratio (Figures 5D–G).

### Protein Kinase C Is a Potential Target of D-Glucono-1,5-Lactone by Bioinformatics Analysis and Molecular Docking Studies

To further understand how GDL regulates ERK signaling, we used Swiss Target Prediction databases^2^ to sort out the potential targets of GDL. Species were selected as “Homo sapiens,” and a total of 23 targets were obtained under the condition of probability greater than 0.1 (Supplementary Table 1). Among them, Protein kinase C (PKC) isoforms, including PKCδ, PKCα, PKCγ, PKCε, PKCη, PKCθ, stood out as the most possible target for GDL.

**FIGURE 6:**
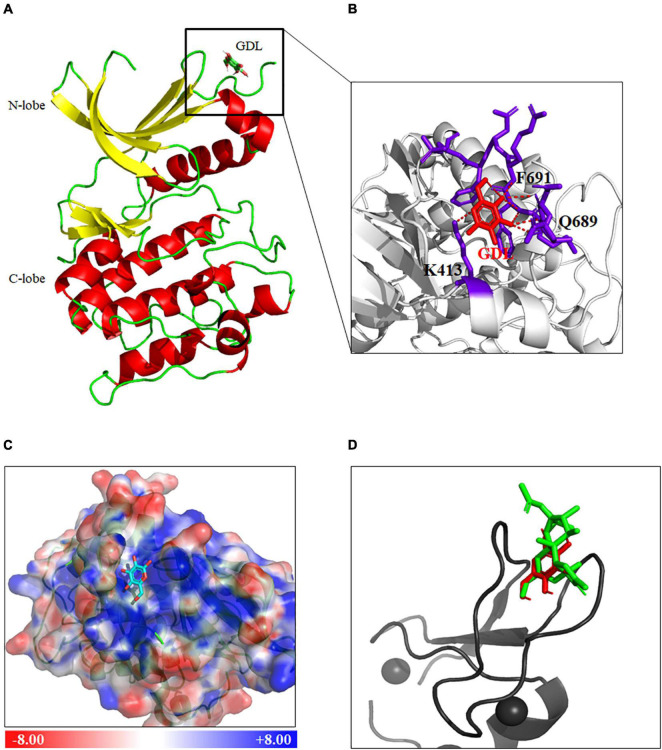
Protein kinase C is a putative target of GDL by bioinformatics analysis and molecular docking studies. **(A)** Docking of GDL into PKC (PDB code 1XJD) by AutoDock Vina, and the protein-ligand interaction of PKC and GDL was visualized by PyMOL (www.pymol.org). **(B)** Formation of hydrogen bonds between GDL and PKC (Red, GDL). **(C)** Electrostatic surface of PKC structure generated by APBS tool (Blue, positive charge; red, negative charge; Cyan, GDL). **(D)** Docking of GDL into PKC (PDB code 1PTR) by AutoDock Vina, and comparison of docking poses in PKC between Phorbol-13-acetate (Green) and GDL (Red).

Next, Autodock Vina was used to predict the binding modes of GDL with PKC (Trott and Olson, 2009). The crystal structure of PKC (PDB code 1XJD) was obtained from the Protein Data Bank (PDB), and GDL was docked into PKC structure by Autodock Vina. Among twenty conformation generated, the best one has been nominated based on lowest binding energy (-5.0 Kcal/mol). Protein-ligand interaction of docked complex was visualized by PyMOL software^3^ (Figure 6A). The hydrogen bonds were analyzed by PyMOL software, showing GDL interacts with PKC by forming hydrogen bonds with Q688, F691, and K413 (Figure 6B). Electrostatic surface of PKC was generated by APBS tool, showing GDL buried in a positive charged pocket of PKC (Figure 6C).

The NMR structure of PKC binding with Phorbol-13-acetate (PDB code 1PTR) has been revealed before, thus, we try to compare the binding poses of GDL with Phorbol-13-acetate. GDL was docked into the crystal structure of PKCδ C1 domain by Autodock Vina (binding energy -5.0 Kcal/mol), and we can see GDL occupied the same position in PKC structure as Phorbol-13-acetate after structural alignment (Figure 6D).

### Protein Kinase C Isoform PKCε Is Considered as the Direct Target of D-Glucono-1,5-Lactone by Functional Identification

**FIGURE 7:**
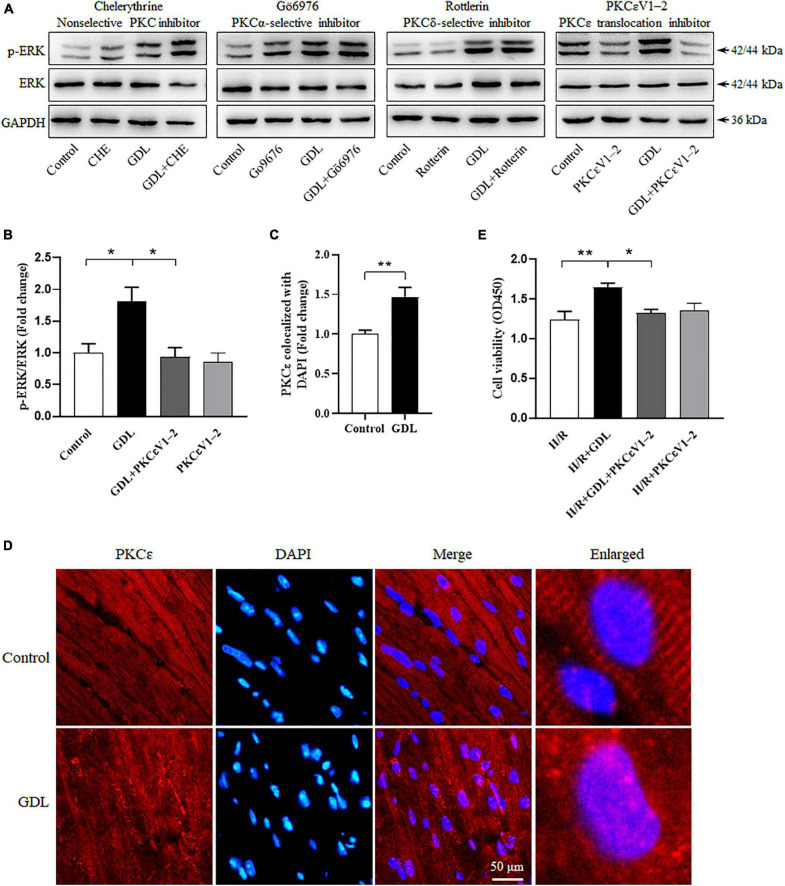
Protein kinase C isoform PKCε is identified as the direct target of GDL by functional analysis. **(A)** Representative Western blot images of ERK expression and phosphorylation in H/R-injured NRCM cells following the treatment of PKC inhibitors (Chelerythrine, Gö6976, Rottlerin or PKCεV1-2). **(B)** Quantification analysis of ERK phosphorylation in H/R-injured NRCM cells following PKCεV1-2 treatment. **(C,D)** Immunofluorescence staining of PKCε nuclear translocation in the heart of mice treated with or without GDL. **(E)** The effect of PKCεV1-2 on GDL-induced cardioprotection against H/R injury. *P < 0.05 and **P < 0.01 between indicated groups. Data shown are representive of five independent experiments.

Protein kinase C family falls into three groups: the classical PKC (cPKC; α, βI, βII, and γ), atypical PKC (aPKC; ζ and λ/ι) or novel PKC (nPKC; δ, ε, η, θ, μ, and υ). To investigate whether or which PKC isoform contributes to GDL-induced ERK phosphorylation and cardioprotection, we used different PKC inhibitors, including Chelerythrine (a general PKC inhibitor), Gö6976 (an inhibitor of cPKC), Rottlerin (an inhibitor of PKCδ), and PKCεV1-2 (a specific inhibitor of PKCε). Western blot analysis showed that Chelerythrine (5 μM), Gö6976 (1 μM), and Rottlerin (25 μM) had no effect on GDL-induced ERK phosphorylation, whereas PKCε specific inhibitor PKCεV1-2 (20 μM) attenuated this effect (Figures 7A,B). Immunofluorescence staining showed that i.p. administration of GDL in mice increased PKC**ε** translocation into nuclear (Figures 7C,D). Cell viability assay showed that PKCεV1-2 attenuated GDL-conferred cardioprotection against H/R injury (Figure 7E).

## Discussion

**FIGURE 8:**
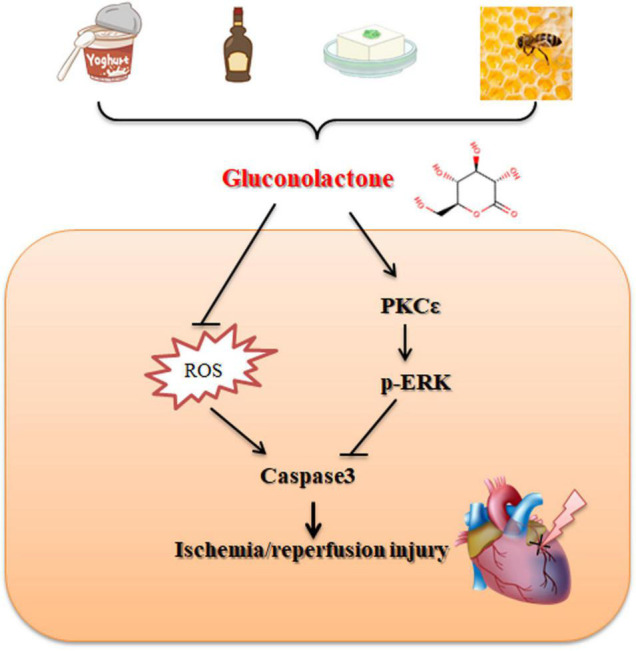
Schematic illustrating the cardioprotective effect of GDL against I/R injury.

D-glucono-1,5-lactone is a widely used food additive and presents in a variety of natural products and fermented food. However, its roles in cardiovascular diseases remain elusive. In this study, using a mouse model of I/R injury and a cellular model of H/R mimicking this process, we revealed that GDL administration (5 mg/kg, i.p.) attenuated I/R injury and reperfusion-induced arrhythmias, and pharmacological inhibition of ERK signaling attenuated GDL-conferred cardioprotection. Bioinformatics analysis and molecular docking studies revealed that PKC is a potential target for GDL, and subsequent functional analysis demonstrated PKC isoform PKCε as its direct target. Taken together, our results demonstrated that natural product GDL acts as a direct activator of PKCε and, thus, provides cardioprotection against I/R injury *via* activation of ERK signaling (Figure 8).

In human body, besides derived from foods, GDL is also a metabolite of gut microbiota, thus having the capacity to affect physiological and pathophysiological processes when the intestinal homeostasis is altered. Zhao et al. (2018) demonstrated that fecal GDL increased about twofold in normal human subjects after half-marathon race compared with those before race. A datasets of caecal metabolites of normal diet and high-fat-diet (HFD) mice (MTBLS545) available from Metabolights^4^ was analyzed and profiled, demonstrating an increase of caecal GDL levels in HFD-induced obese mice (Supplementary Figure 1A; Zheng et al., 2017). Of note, another dataset of metabolomics of mice inoculated with human microbiota available from Metabolomics Workbench (ST000745^5^) showed that an increase of GDL levels in peripheral blood at 35 days post-inoculation of human microbiota compared with that at 6 days (Supplementary Figure 1B). These results demonstrated that GDL might be a critical mediator between gut microbiota and human health.

D-glucono-1,5-lactone is known as a potent antioxidant mainly for two reasons: (1) GDL is a natural PHA, which is capable of chelating metals and may function as a free radical scavenger (Bernstein et al., 2004); (2) GDL regulates redox balance *via* entering into the PPP pathway (Wushensky et al., 2018). Supportively, we found that GDL treatment decreased oxidative stress in reperfusion injured mice, which is probably due to the increase of NADPH production and the decrease of ROS production (Figure 3). NADPH is a major component to detoxify ROS by reducing glutathione, and decreased NADPH is associated with the loss of antioxidant activity and the increase of ROS production. Our results suggested that GDL increased NADPH/NADP^+^ ratio in cardiomyocytes from I/R-injured mice, thereby enhancing cellular ROS-scavenging capacity. However, the detailed mechanism how GDL increased NADPH production needs further elucidation.

MEK/ERK signaling and PI3K/Akt signaling are two major components of the pro-survival reperfusion injury salvage kinase (RISK) signaling pathway. Our results demonstrated that GDL exerts cardioprotection *via* ERK but not Akt signaling. Our *in vivo* experiments showed that GDL treatment activated ERK but not Akt signaling in mice subjected to I/R (Figure 1F). Consistently, GDL treatment increased ERK signaling in H/R-injured NRCM cells (Figure 4D). In addition, GDL treatment activated ERK in a time-dependent manner, reaching a peak value at 2 h post treatment (Figure 5B). However, our results also showed that high concentration of GDL (>10 μM) is toxic for cardiomyocytes (Figure 4A), probably *via* different mechanisms.

Extracellular signal-regulated kinase signaling was highly associated with the biological effects of lactone, yet the actual effects (inhibitory or activated) of lactones on ERK signaling is inconsistent depending on the lactone type and cell type. GDL is a δ-lactone, and we showed that GDL increased ERK phosphorylation *in vivo* and *in vitro* (Figures 1, 4, 5). In line with our results, Emblicanin A and Emblicanin B, δ-lactones from *Emblica officinalis*, up-regulated ERK signaling (Sumitra et al., 2009). Macro-cyclic lactone bryostatin1 activated PKC and downstream ERK in many cell types (Zhang and Liu, 2002). However, Goniothalamin and Rasfonin, also members of δ-lactone, were found to inhibit ERK phosphorylation in different cancer cells (Xiao et al., 2014; Innajak et al., 2016). Moreover, the effects of sesquiterpene lactones on ERK signaling were widely explored, showing different effects following different sesquiterpene lactone treatment (Kim et al., 2005, 2008; Michalak et al., 2019).

Protein kinase C is involved in a variety of cellular processes including cell proliferation, differentiation, hypotrophy, apoptosis, and modulates various signaling pathways including ERK signaling pathway (Singh et al., 2017). Herein, Swiss target prediction showed that PKC isoforms are potential targets of GDL, which was further confirmed by functional identification. Subsequently, the interaction of PKC with GDL was simulated by using molecular docking Autodock Vina, demonstrating that GDL could bind to the C1 domain of PKC, resulting in its activation, which is consistent with other PKC activators DAG or phorbol ester (Zhang et al., 1995). Of note, it has been reported that diacylglycerol-lactone 130C037 has different affinity for PKC isoforms (higher affinity for PKCδ and lower affinity for PKCα) (Pu et al., 2005), showing its binding preference to PKC isoforms. Likewise, functional analysis showed that GDL activated ERK signaling mainly *via* PKCε but not other PKC isoforms (Figure 7). In the context of myocardial I/R injury, PKC isoforms are believed to have distinct roles in regulating reperfusion injury (Budas et al., 2007; Chen et al., 2021). Generally speaking, inhibition of PKCα, PKCβ, and PKCδ attenuated I/R injury, while activation of PKCε protects the heart against reperfusion injury (Budas et al., 2007; Kong et al., 2008). In this study, we found that GDL stimulated ERK phosphorylation and resultant cardioprotection mainly *via* activating PKCε (Figure 7). These data suggested that GDL has a clinical potential as a cardioprotective drug against I/R injury.

Both bioinformatics analysis and functional identification showed GDL is a potent activator of PKCε, thereby exerts cardioprotection against I/R injury; however, direct biochemical evidence for the binding of GDL to PKCε is still limited, and needs further exploration.

## Conclusion

Taken together, our results demonstrated that a natural metabolite GDL provides cardioprotection against myocardial I/R injury *via* targeting PKC and subsequent activation of ERK signaling.

## Data Availability Statement

The datasets presented in this study can be found in online repositories. The names of the repository/repositories and accession number(s) can be found in the article/Supplementary Material.

## Ethics Statement

The animal study was reviewed and approved by the Animal Care Committee of Northwestern Polytechnical University.

## Author Contributions

XZ, JX, and XQ took the responsibility for study design. XQ, BL, and FG performed the experiments and data acquisition. XQ, JX, ZC, and YH analyzed the data. XQ and XZ wrote the manuscript. All authors contributed to manuscript revision, read, and approved the submitted version.

## Conflict of Interest

The authors declare that the research was conducted in the absence of any commercial or financial relationships that could be construed as a potential conflict of interest.

## Publisher’s Note

All claims expressed in this article are solely those of the authors and do not necessarily represent those of their affiliated organizations, or those of the publisher, the editors and the reviewers. Any product that may be evaluated in this article, or claim that may be made by its manufacturer, is not guaranteed or endorsed by the publisher.

## Footnotes

1
https://imagej.net/Coloc2

2
http://swisstargetprediction.ch/

3
www.pymol.org

4
https://www.ebi.ac.uk/metabolights/index

5
https://www.metabolomicsworkbench.org/

## Abbreviations

AAR: area at risk
CHE: Chelerythrine
DHE: Dihydroethidium
ECG: electrocardiogram
ERK: extracellular signal-regulated kinase
GDL: D-glucono-1,5-lactone
HR: heart rate
H/R: hypoxia/reoxygenation
I/R: ischemia/reperfusion
LDH: lactate dehydrogenase
LV: left ventricle
MDA: malondialdehyde
NRF2: the nuclear factor erythroid 2 related factor 2
PHA: polyhydroxy acid
PKC: protein kinase C
PVC: premature ventricular contraction
ROS: reactive oxygen species
SOD: superoxide dismutase
TUNEL: the terminal deoxynucleotidyl transferase (TdT) dUTP nick-end labeling
VF: ventricular fibrillation
VT: ventricular tachycardia.
