# Supplementary material for: Gluconolactone Alleviates Myocardial Ischemia/Reperfusion Injury and Arrhythmias via Activating PKCε/Extracellular Signal-Regulated Kinase Signaling
Source: Front Physiol. 2022 Mar 14;13:856699. doi: 10.3389/fphys.2022.856699 (PMC8964113; doi:10.3389/fphys.2022.856699)
Supplement: Supplementary file 1 [file Image_1.pdf]

Supplementary Material

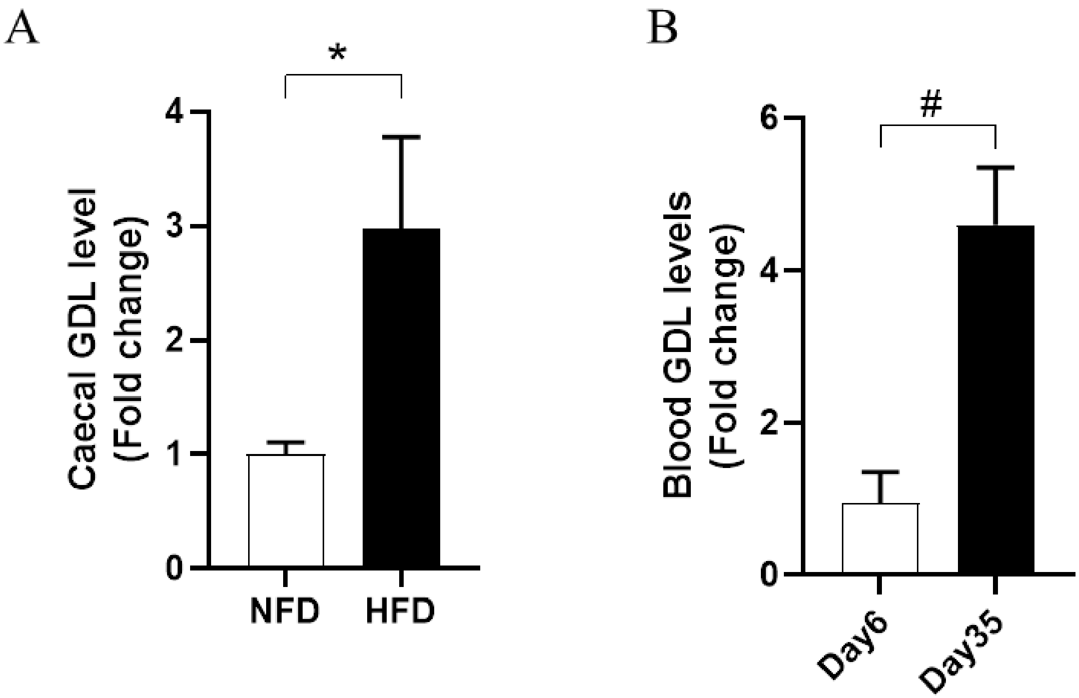

Supplementary Figure 1. GDL is an important metabolite of gut microbiota. (A) Apparent increase of caecal GDL levels in high-fat-diet (HFD)-induced obese mice. Data were available from MetaboLights Repository, https://www.ebi.ac.uk/metabolights/MTBLS545. (B) Increase of GDL levels in peripheral blood at 35 days post-inoculation of human microbiota compared with that at 6 days. This data is available at the NIH Common Fund's National Metabolomics Data Repository (NMDR) website, the Metabolomics Workbench, https://www.metabolomicsworkbench.org where it has been assigned Project ID PR000538. The data can be accessed directly via its Project DOI: 10.21228/M84687. This work is supported by NIH grant U2C-DK119886.
