# Supplementary material for: Gluconolactone Alleviates Myocardial Ischemia/Reperfusion Injury and Arrhythmias via Activating PKCε/Extracellular Signal-Regulated Kinase Signaling
Source: Front Physiol. 2022 Mar 14;13:856699. doi: 10.3389/fphys.2022.856699 (PMC8964113; doi:10.3389/fphys.2022.856699)
Supplement: Supplementary file 2 [file Table_1.pdf]

Supplementary Material

Supplementary Table 1 Potential targets of GDL by Swiss Target Prediction

Number	Target	Common name	Uniprot ID	ChEMBL ID	Target Class	Probability*	Known actives (3D/2D)
1	Protein kinase C delta (by homology)	PRKCD	Q05655	CHEMBL2996	Kinase	0.133391	0/19
2	Protein kinase C alpha	PRKCA	P17252	CHEMBL299	Kinase	0.133391	0 /105
3	Brain glycogen phosphorylase	PYGB	P11216	CHEMBL3856	Enzyme	0.133391	3/0
4	HMG-CoA reductase (by homology)	HMGCR	P04035	CHEMBL402	Oxidoreductase	0.125076	0/41
5	Transient receptor potential cation channel subfamily V member 4 (by homology)	TRPV4	Q9HBA0	CHEMBL3119	Voltage-gated ion channel	0.125076	0/5
6	Liver glycogen phosphorylase	PYGL	P06737	CHEMBL2568	Enzyme	0.125076	3/0
7	NAD-dependent deacetylase sirtuin 2	SIRT2	Q8IXJ6	CHEMBL4462	Eraser	0.125076	0/1

8	Muscle glycogen phosphorylase	PYGM	P11217	CHEMBL3526	Enzyme	0.125076	6/0
9	P-glycoprotein 1	ABCB1	P08183	CHEMBL4302	Primary active transporter	0.125076	0/33
10	Kappa Opioid receptor	OPRK1	P41145	CHEMBL237	Family A G protein-coupled receptor	0.125076	0/18
11	Protein kinase C gamma (by homology)	PRKCG	P05129	CHEMBL2938	Kinase	0.125076	0/10
12	Protein kinase C epsilon	PRKCE	Q02156	CHEMBL3582	Kinase	0.125076	0/18
13	Protein kinase C eta (by homology)	PRKCH	P24723	CHEMBL3616	Kinase	0.125076	0/9
14	Protein kinase C theta	PRKCQ	Q04759	CHEMBL3920	Kinase	0.125076	0/11
15	Gamma-secretase	PSEN2<br>PSENEN<br>NCSTN<br>APH1A<br>PSEN1<br>APH1B	P49810<br>Q9NZ42<br>Q92542<br>Q96BI3<br>P49768<br>Q8WW43	CHEMBL2094135	Protease	0.125076	0/16
16	11-beta-hydroxysteroid dehydrogenase 1	HSD11B1	P28845	CHEMBL4235	Enzyme	0.125076	0/9

17	Platelet activating factor receptor (by homology)	PTAFR	P25105	CHEMBL250	Family A G protein-coupled receptor	0.116739	0/6
18	Mu opioid receptor	OPRM1	P35372	CHEMBL233	Family A G protein-coupled receptor	0.116739	0/3
19	Glucose transporter	SLC2A1	P11166	CHEMBL2535	Electrochemical transporter	0.116739	0/1
20	Brain adenylate cyclase 1	ADCY1	Q08828	CHEMBL2899	Enzyme	0.116739	0/31
21	Vitamin D receptor	VDR	P11473	CHEMBL1977	Nuclear receptor	0.116739	0/2
22	Carbonic anhydrase I	CA1	P00915	CHEMBL261	Lyase	0.116739	6/8
23	Carbonic anhydrase IX	CA9	Q16790	CHEMBL3594	Lyase	0.116739	11/8
